# EHMTI-0385. Dynamic ultrasound of the optic nerve sheath diameter in patients with orthostatic headaches: a new diagnostic tool in patients with spontaneous intracranial hypotension

**DOI:** 10.1186/1129-2377-15-S1-J4

**Published:** 2014-09-18

**Authors:** J Beck, J Fichtner, CT Ulrich, C Fung, P Schucht, A Jilch, A Raabe

**Affiliations:** 1 Neurosurgery, University of Bern, Bern, Switzerland

## Objective

Spontaneous intracranial hypotension (SIH) caused by cerebrospinal fluid leakage commonly presents with orthostatic headache. We hypothesize that positional changes, i.e. a decrease of the optic nerve sheath diameter (ONSD) occur from supine to upright position in symptomatic patients with orthostatic headaches. We performed an ultrasound study investigating whether there are positional changes in ONSD in symptomatic patients suffering orthostatic headaches.

## Methods

Dynamic ultrasound was performed in 44 consecutive patients with suspected SIH. In 18 patients the leading symptom was orthostatic headaches (Group A:10 men, 8 women; mean age 51.9 years), while 26 patients did not suffer from acute orthostatic headaches (Group B: 15 men, 11 women; mean age 61.9 years).

## Results

In supine position ONSD were similar in both groups (A: mean 0.538 vs. B: 0.539cm; p=0.957). In the upright position mean ONSD was significantly smaller in patients with orthostatic headaches (mean 0.484 ± SD 0.095cm) as compared to patients without (0.549 ± SD 0.097cm, p=0.036). Patients with orthostatic headaches showed a larger change of ONSD from supine to upright position (mean -0.053 ± SD 0.034cm) compared to patients without orthostatic headaches (0.005 ± SD 0.038cm, p≤0.001).

## Conclusion

In this series significant changes of ONSD occurred during dynamic measurement from supine to upright patient position only in patients with acute orthostatic headaches. We call this method of comparing supine and subsequent upright ONSD “dynamic assessment of the optic nerve sheath diameter by ultrasound”. Transorbital dynamic ultrasound may become a useful, novel, non-invasive diagnostic tool for patients with orthostatic headaches.

No conflict of interest.

